# Commentary: Current perspectives and trends in colorectal cancer and cancer-associated fibroblasts: a review and bibliometric analysis

**DOI:** 10.3389/fimmu.2025.1672250

**Published:** 2026-01-07

**Authors:** Hua Zhao

**Affiliations:** Hubei University of Chinese Medicine Affiliated Gong’an Hospital of Traditional Chinese Medicine, Jingzhou, China

**Keywords:** bibliometrics, cancer-associated fibroblasts, colorectal cancer, trends, tumor microenvironment

## 1 Introduction

The burgeoning volume of biomedical literature in recent years has significantly heightened academic interest in bibliometrics as a methodological tool. This approach enables the quantitative and qualitative analysis of research trajectories and emerging focal points within specific disciplines. We read with considerable interest the article by Qian, Chengyong et al. (1), entitled “Current perspectives and trends in colorectal cancer and cancer-associated fibroblasts: a review and bibliometric analysis,” published in Frontiers in Immunology. Utilizing bibliometric techniques, this study provides a systematic mapping of the global research landscape, identifying leading contributing countries, institutions, and researchers in the field of colorectal cancer (CRC) and cancer-associated fibroblasts (CAFs). It underscores that investigating the tumor-promoting mechanisms of CAFs and developing precise targeted therapies against CAFs represent prominent current research hotspots. Furthermore, the analysis suggests that the long-term prognosis of patients undergoing such targeted therapies may emerge as a critical focus for future inquiry. We highly support and appreciate the researchers’work, and thank them for their contributions in the field. However, we identified several points requiring clarification and correction.

First, regarding Journal Publication and Citation Metrics: The abstract states: “CANCERS has the most publications, and the highest citation rate.” While **Table 1** confirms that CANCERS published the most articles in the field (N = 48), its average citation rate (3.15) is substantially lower than that of the INTERNATIONAL JOURNAL OF CANCER (14.47). Therefore, based on the provided data, the INTERNATIONAL JOURNAL OF CANCER demonstrates the highest citation rate, contradicting the claim in the abstract.

**Table 1:** Leading journals in zone one for colorectal cancer and cancer-associated fibroblasts studies ranked by issuance and citations.

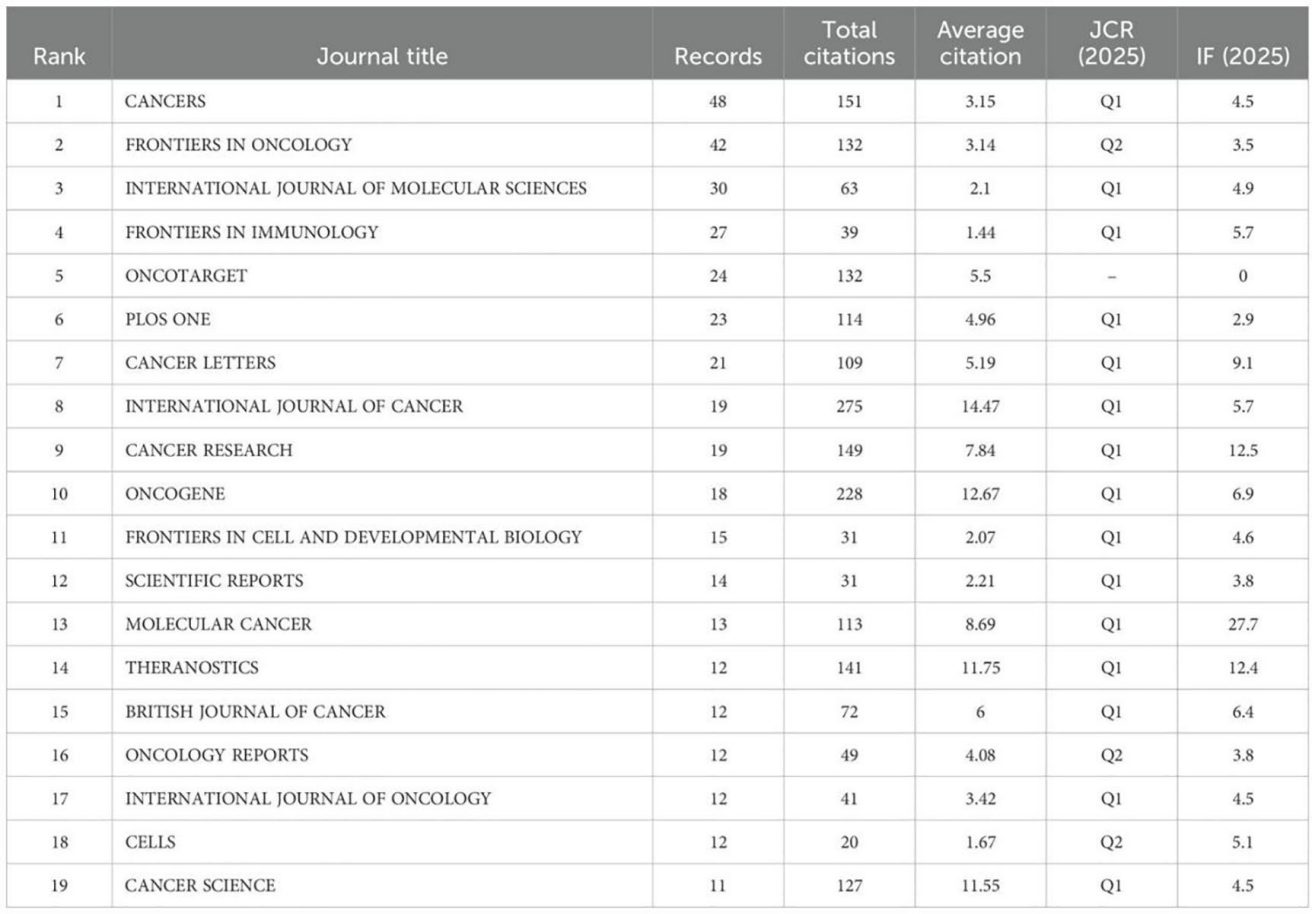

**Figure 3:**
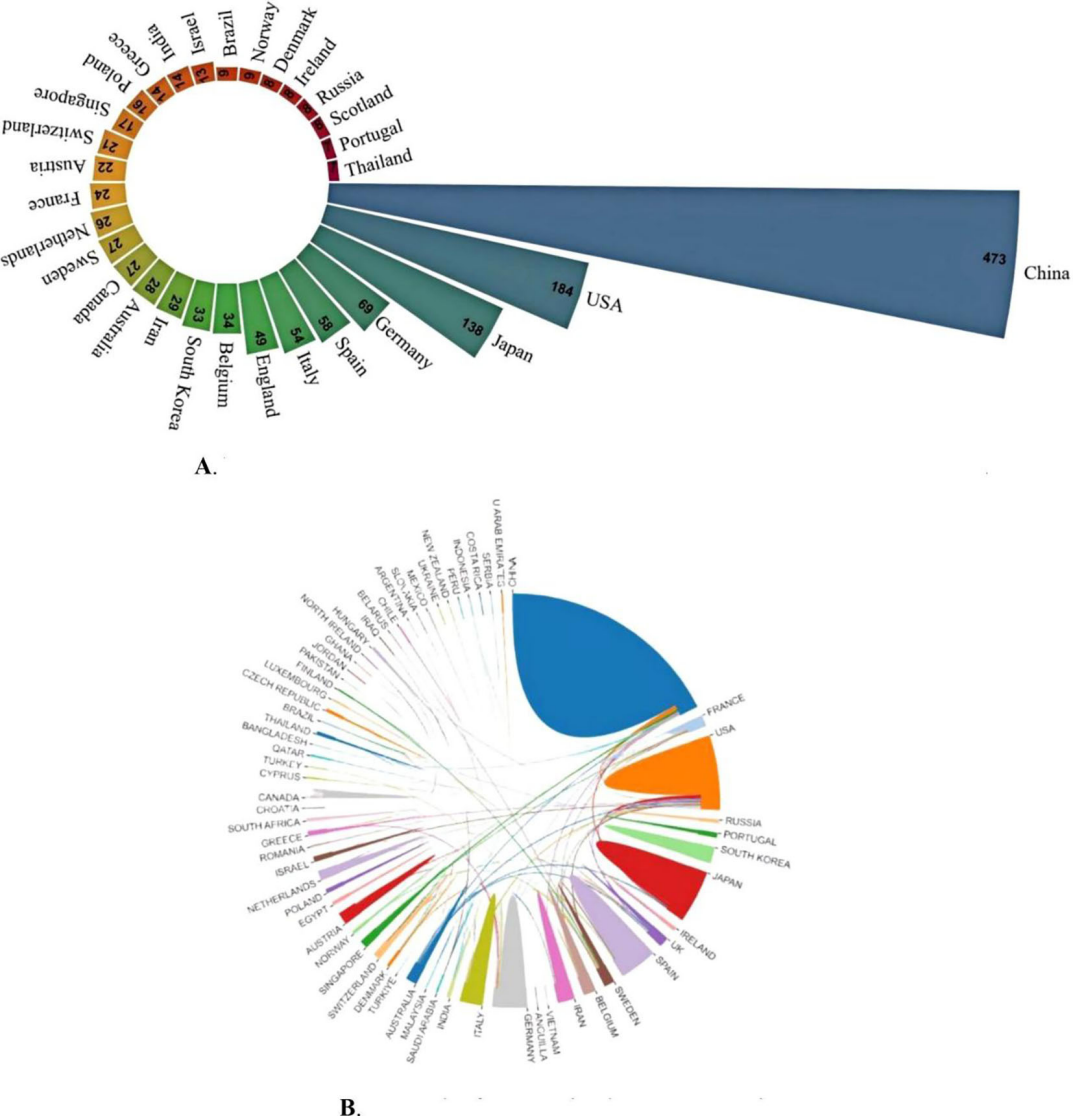
**(A)** Nightingale’s Rose diagram displaying publications from the top 30 countries. **(B)** Network of cooperation between countries.

Second, regarding Country/Region and Institution Analysis: the section “Situation of countries/regions and institutions” states: “The United States and Japan are next (N = 184).” However, **Figure 3** clearly shows the USA with 184 publications and Japan with only 138 publications. Attributing the count of 184 jointly to both countries is inaccurate. Furthermore, the same section states: “in terms of total and average counts of citations, it trails behind Fudan University and the Medical University of Vienna.” This is inconsistent with **Table 3**, which shows that despite publishing fewer articles (N = 37), Leiden University ranks highest in both total citations (489) and average citations (13.22) among the listed institutions. The significant contribution of Leiden University based on citation impact warrants greater emphasis.

**Table 3:** Leading journals in zone one for colorectal cancer and cancer-associated fibroblasts studies ranked by issuance and citations.

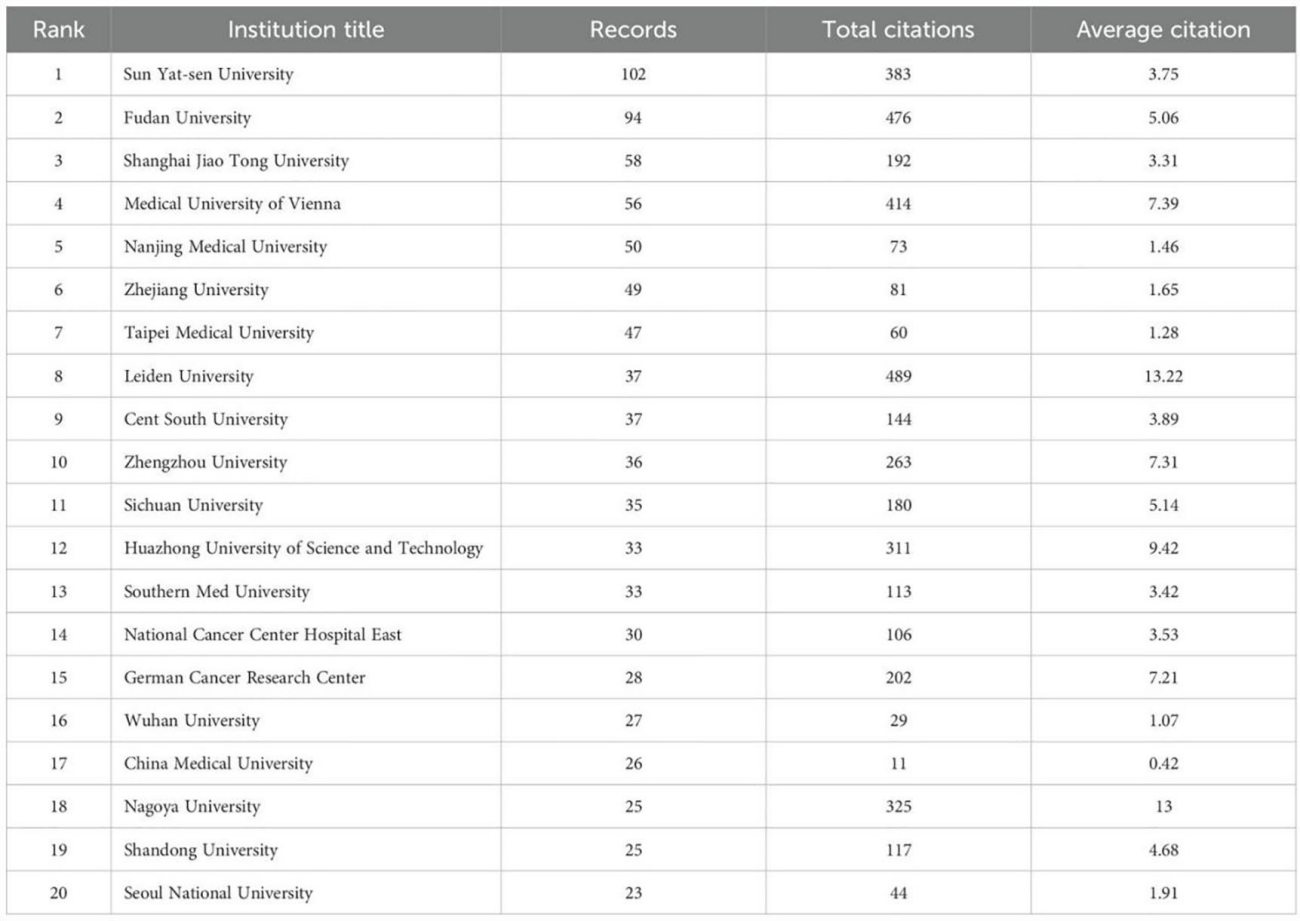

**Figure 6:**
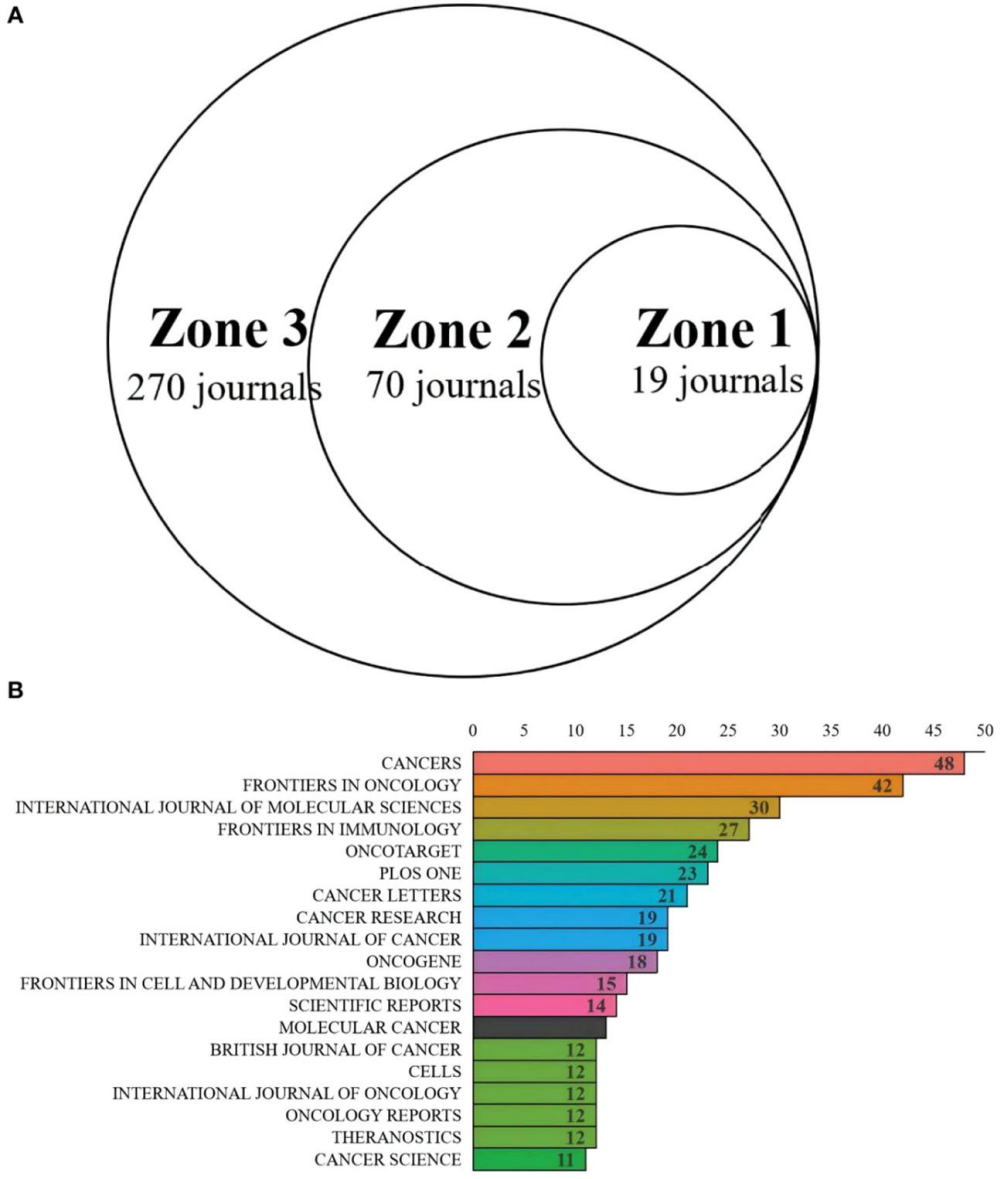
**(A)** Journal productivity analysis according to Bradford’s law. **(B)** Distribution map of journal publications in zone one.

Third, regarding Journal Analysis: the “Journals analysis” section states: “The Zones 1 and 2 each contained 19 journals.” This is contradicted by **Figure 6A**, which depicts Zone 1 containing 19 journals and Zone 2 containing 70 journals. Subsequently, the text ranks journals by publication count: “CANCERS is ranked first (N = 48), followed by FRONTIERS IN ONCOLOGY (N = 42), the INTERNATIONAL JOURNAL of MOLECULAR RESEARCH (N = 30).” **Figure 6B**, however, identifies the INTERNATIONAL JOURNAL OF MOLECULAR SCIENCES (not “Research”) as the third-ranked journal by publication volume.

Finally, we commend the authors for their valuable contribution, which offers strategic insights for researchers and helps delineate potential directions for subsequent studies on CRC and CAFs. Notwithstanding these strengths, we have identified several aspects within the manuscript that, in our view, would benefit from further clarification and correction to enhance the study’s rigor. To ensure the utmost scientific precision and scholarly rigor of the published work, we believe a more meticulous formulation in certain sections would be advantageous.
